# Multidrug resistant and extensively drug resistant *Acinetobacter baumannii* hospital infection associated with high mortality: a retrospective study in the pediatric intensive care unit

**DOI:** 10.1186/s12879-020-05321-y

**Published:** 2020-08-12

**Authors:** Jingyi Shi, Ting Sun, Yun Cui, Chunxia Wang, Fei Wang, Yiping Zhou, Huijie Miao, Yijun Shan, Yucai Zhang

**Affiliations:** 1 Department of Critical Care Medicine, Shanghai Children’s Hospital, Shanghai Jiao Tong University, Shanghai, 20062 China; 2 Institute of Pediatric Critical Care, Shanghai Jiao Tong University, Shanghai, 20062 China; 3 Department of Critical Care Medicine, Shanghai, Children’s Hospital, Shanghai Jiao Tong University, No.355 Luding Road, Putuo District, Shanghai, 200062 China

**Keywords:** MDR/XDR, *Acinetobacter baumannii*, Risk factors, Mortality, Pediatric intensive care units

## Abstract

**Background:**

Multidrug resistant (MDR) and extensively drug resistant (XDR) *Acinetobacter baumannii* presents challenges for clinical treatment and causes high mortality in children. We aimed to assess the risk factors and overall mortality for MDR/XDR *Acinetobacter baumannii* infected pediatric patients.

**Methods:**

This retrospective study included 102 pediatric patients who developed MDR/XDR *Acinetobacter baumannii* infection in the pediatric intensive care unit (PICU) of Shanghai Children’s Hospital in China from December 2014 to May 2018.

*Acinetobacter baumannii* clinical isolates were recovered from different specimens including blood, sputum, bronchoalveolar lavage fluid, cerebrospinal fluid, ascites, hydrothorax, and urine. Antibiotic susceptibility test was determined according to the Clinical and Laboratory Standards Institute interpretive criteria. Clinical and biological data were obtained from the patients’ medical records.

**Results:**

102 patients with *Acinetobacter baumannii* infection were enrolled. The median age was 36 (9.6, 98.8) months, and there were 63 male in the case group. The overall mortality rate was 29.4%, while the *Acinetobacter baumannii*-associated mortality rate was 16.7% (17/102, 12 bloodstream infections, 4 meningitis and 1 intra-abdominal infection). Bloodstream infections occurred in 28 patients (27.5%), and 10 patients (9.8%) among them had central line-associated bloodstream infections (6 central venous catheters, 2 PICCs, 1 venous infusion port and 1 arterial catheter). Cerebrospinal fluid (CSF) cultures were positive in 4(3.9%) patients. 14(13.7%) patients got positive cultures in ascites and hydrothorax. Lower respiratory isolates (56/102) accounted for 54.9% of all patients. Non-survival patients appeared to have a lower NK cell activity (6.2% ± 3.61% vs. 9.15% ± 6.21%, *P* = 0.029), higher CD4+ T cell ratio (39.67% ± 12.18% vs. 32.66% ± 11.44%, *P* = 0.039),and a higher serum level of interlukin-8 (IL-8, 15.25 (1.62, 47.22)pg/mL vs. 0.1 (0.1, 22.99)pg/mL, *P* = 0.01) when *Acinetobacter baumannii* infection developed. Multivariate logistic analysis indicated that high serum level of Cr (RR, 0.934, 95%CI, 0.890–0.981; *P* = 0.007) and high BUN/ALB level (RR, 107.893, 95%CI, 1.425–870.574; *p* = 0.005) were associated with high risk of mortality in MDR/XDR *Acinetobacter baumannii* infected patients.

**Conclusion:**

MDR/XDR *Acinetobacter baumannii* infection is a serious concern in pediatric patients with high mortality. Bloodstream and central nervous system infection accounted for high risk of death. Acute kidney injury is associated with high risk of mortality.

## Background

*Acinetobacter baumannii* is a Gram-negative coccobacillus that has a remarkable ability to acquire antibiotic resistance and that cause persistent nosocomial infections [1] The mortality rate varies from 18.26 to 88.7% depending on the infection source [2, 3]. The propensity of *Acinetobacter baumannii* to be multidrug-resistant (MDR) or extensively drug-resistant (XDR) presents therapeutic challenges [4, 5]. Invasive operations such as endotracheal mechanical ventilation, inserted invasive devices, intensive care unit stay, recent surgery, use of broad-spectrum antibiotics, ineffective management, and septic shock at diagnosis are reported as risk factors for colonization or infection by MDR *Acinetobacter baumannii* and higher mortality [6, 7].

The incidence of infections due to MDR *Acinetobacter baumannii* in Southeast Asia were higher compared to other areas [8, 9], According to a five-year case-control study from Southwest China, it was the severity of illness (high APACHE II score and MODS) that highlighted the mortality of patients with nosocomial *Acinetobacter baumannii* bacteremia [10]. The problems for pediatrician in China are polymyxins cannot be obtained, tigecycline only became available in late 2016, and had limited experience in manage these young patients [11, 12].

In pediatric critically ill patients, underlying diseases, immune deficiency, and invasive operations all contributed to the higher incidence of multidrug resistant *Acinetobacter baumannii* infection [13]. Multidrug resistant *Acinetobacter baumannii* infection is responsible for a high mortality rate among neonates in the NICU [14]. According to a report from Seoul National University Children’s Hospital, carbapenem nonsusceptibility, neutropenia, and prolonged ICU stay as independent risk factors for mortality due to *Acinetobacter baumannii* bacteremia in PICU patients [15]. However, no study has examined these risk factors for mortality or clinical features due to MDR/XDR *Acinetobacter baumannii* infection in critically ill pediatric patients in China. Thus, the objective of this study was to assess the outcome and risk factor in hospitalized children with multidrug resistant and extensively drug resistant *Acinetobacter baumannii* infection in a pediatric intensive care unit.

## Methods

This retrospective study included pediatric patients who developed multidrug resistant and extensively drug resistant *Acinetobacter baumannii* infection in the pediatric intensive care unit (PICU) of Shanghai Children’s Hospital in China from December 2014 to May 2018. Colonization was defined as positive culture results from clinical isolated samples (sputum and urethral catheter tips) without clinical manifestation of infection.

*Acinetobacter baumannii* clinical isolates were recovered from different specimens including blood, sputum, bronchoalveolar lavage fluid, cerebrospinal fluid, ascites, hydrothorax, wound swabs, and urine from immunocompromised pediatric patients with symptomatic clinical infections at least 48 h after PICU admission. Specimens collected from from different specimens were placed in 3 mL BHI broth and incubated at 37 °C with constant rotation for 24 h and were then spread on MacConkey agar. An aliquot of 5 mL nutrient broth was homogenized added to agar, and incubated for 5 min at 22–25 °C. Then spread 100 μL of sample on blood agar and incubated at 37 °C for 24 h. After that, a volume of 100 μL saline peptone obtained from each floor sample was spread on blood agar and incubated at 37 °C for 24 h. Colonies were preliminarily screened by morphology and characterized by oxidase and triple sugar iron tests. The antibiotic susceptibility was determined using the disk diffusion method in our study. The following antimicrobial disks were used: ampicillin-sulbactam (10/10 μg), ceftazidime (30 μg), imipenem (10 μg), gentamicin (10 μg), co-trimoxazole (1.25/23.75 μg), piperacillin (100 μg) and cefepime (30 μg). Susceptibility testing to the tested antibiotics was determined according to the Clinical and Laboratory Standards Institute (CLSI) interpretive criteria for disk diffusion method, as well as the broth dilution MIC measurement [16]. Serial two-fold dilutions ranging from 256 to 0.25 mg/L for ceftazidime, meropenem, and from 128 to 0.015 mg/L for tigecycline and colistin were prepared in fresh 96-well microtiter plates. The inoculum was prepared with a 6-h broth culture that gives a final concentration of ~ 105 CFU/ml in the test tray. The inoculums were then incubated overnight (16–18 h) at 37 °C without shaking. The MIC was defined as the lowest concentration of antibiotic giving complete inhibition of visible growth. MDR was defined as acquired non-susceptibility to at least one agent in three or more antimicrobial categories, XDR was defined as non-susceptibility to at least one agent in all but two or fewer antimicrobial categories (i.e. bacterial isolates remain susceptible to only one or two categories) and PDR (pandrug resistant) was defined as non-susceptibility to all agents in all antimicrobial categories [17]. According to CLSI criteria, we defined infection as follow:1) Blood stream infection: Patient has fever and has a recognized pathogen cultured from 1 or more blood cultures and organism cultured from blood is not related to an infection at another site, or had hemodynamic instability.2) Ventilator associated pneumonia (VAP): Patients with new or progressive and persistent infiltrate in X-rays, with new onset of purulent sputum, change in the character of sputum, more suctioning requirements or worsening gas exchange.3) Central line-associated bloodstream infection: A central line-associated bloodstream infection was defined as a patient who had at least 1 of the following signs or symptoms: hypothermia or hyperthermia, apnea and positive laboratory results not related to an infection at another site that were central venous line-associated with the central venous catheter having been in place at the time of, or within 48 h before, the onset of bacteremia. 4) Meningitis: Meningitis was defined as a patient had organisms cultured from cerebrospinal fluid (CSF), as well as who had at least 2 of the following signs or symptoms with no other recognized cause: headache, dizziness, fever, localizing neurologic signs, changing level of consciousness, or confusion. 5) Intraabdominal infections: Intraabdominal infections must meet at least 1 of the following criteria: Patient had at least 2 of the following signs or symptoms with no other recognized cause: fever, nausea, vomiting, abdominal pain, or jaundice, and the patient had organisms cultured from purulent material from intraabdominal space obtained during a surgical operation or needle aspiration. 6) Urinary tract infection: Urinary tract infection was defined as patient had a positive urine culture, that is, ≥10^5^ microorganisms per cc of urine with no more than 2 species of microorganisms. At least 2 urine cultures with repeated isolation of the same uropathogen (gram negative bacteria or Staphylococcus saprophyticus) with ≥10^2^ colonies/mL in nonvoided specimens. Tracheal aspirate specimens were qualified on a quantification (cfu/mL) from the culture, the colony quantification less than 1×10^3^ cfu/mL was considered as possible colonization.

### Clinical conditions and outcomes

Clinical, biological and treatment data were obtained retrospectively from the patients’ medical records. Underlying illnesses at the time of admission to the PICU were classified and pediatric risk of mortality III (PRISM III) scores were measured at the time of PICU admission. Shock was defined according to FEAST study [18]. The overall mortality was defined as death occurring before discharge. The *Acinetobacter baumannii*-associated mortality was defined as death caused by Multidrug resistant and extensively drug resistant *Acinetobacter baumannii* infection but not other pathogen infection. The primary outcome was overall mortality. Secondary outcomes included the *Acinetobacter baumannii*-associated mortality and the length of hospital stay.

### Statistical analysis

Continuous variables were presented as mean ± standard deviation for normal distribution data and as median (interquartile range) for abnormal distribution data. Comparative analysis was conducted using an independent sample nonparametric test. The chi-square test or Fisher’s exact test was conducted. The Mann-Whitney test was used for continuous variables. Variables showing *p* < 0.05 in a univariate analysis were included in a multivariate analysis, which was performed by stepwise logistic regression. All statistical analyses were performed with the Statistical Package for the Social Sciences, version 17.0 (SPSS Inc., Chicago, IL, USA).

## Results

### Clinical manifestations

**Fig. 1:**
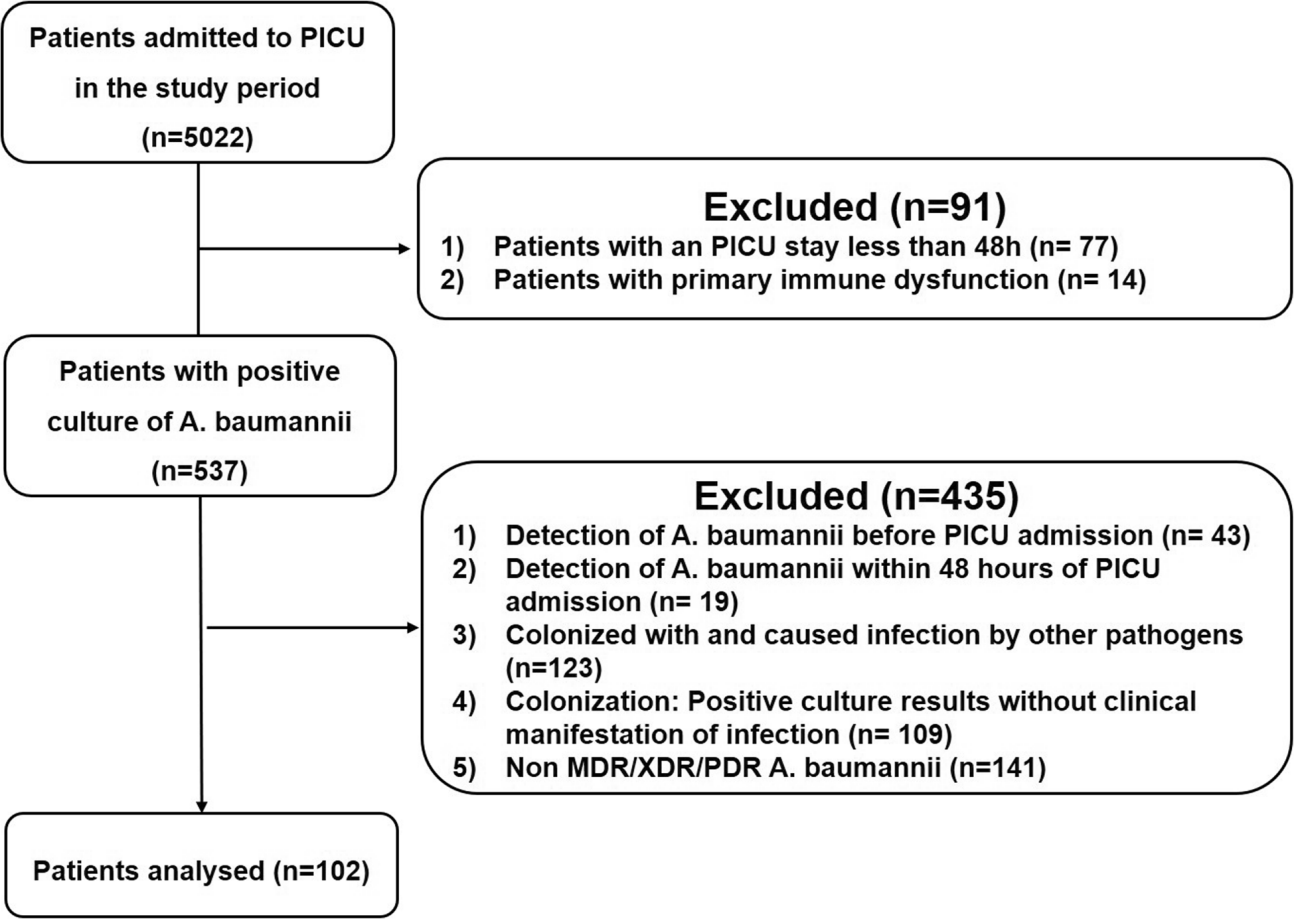
Flowchart of patients with MDR/XDR Acinetobacter baumannii infection enrolled in this study

During the study period of December 1st 2014 and May 31st, 2018, a total of 102 episodes of MDR/XDR *Acinetobacter baumannii* in 102 patients were identified, their demographics and clinical features were summarized in Table 1. The overall incidence of MDR/XDR *Acinetobacter baumannii* was 0.48 cases/1000 patient-days, and we observed a continually increased distribution of cases during the study period. The overall mortality was defined as the survival status when the patient discharged. In our study, the overall mortality was 29.4% (30/102), while the *Acinetobacter baumannii*-associated mortality was 16.7% (17/102). In 28 cases (27.5%) surveillance blood cultures became positive after clinical manifestation appeared, while 10 isolates from catheter (9.8%) (6 central venous catheters, 2 PICCs, 1 venous infusion port and 1 arterial catheter), 4 cerebrospinal fluid (3.9%) and 14(13.7%) ascites and hydrothorax were identified MDR/XDR *Acinetobacter baumannii* positive. 19 (18.6%) samples from tracheal aspirate and 37 (36.3%) from sputum cultures for MDR/XDR *Acinetobacter baumannii* positive. The median age was 36 (9.6, 98.8) months and male predominated (61.8%). 51 patients had surgery (50.0%) and 70 treated with corticosteroids (68.6%), all these patients received broad-spectrum antibiotics treatment for more than one week (Fig. 1. Flowchart of patients with MDR/XDR Acinetobacter baumannii infection enrolled in this study.).

**Table 1:** The Basic Characteristics of MDR/XDR Acinetobacter baumannii patients

Characteristics n (%)		Survivors 72 (70.59)	Nonsurvivors 30 (29.41)	Odds Ratio (95%CI)	*P* value
Age, mean ± SD, month		30 (9.6, 88.75)	42 (9.825, 102.75)	0.691 (−29.63, 12.02)	0.404
Male/Female (n=)		44/28	19/11	(0.286,0.478)	0.835
Underlying disorders	**Pneumonia** n (%)	61 (84.7)	25 (83.3)	0.902 (0.284,2.862)	0.862
	**Meningitis** n (%)	1 (1.38)	6 (20)	17.75 (2.033,154.993)	< 0.001*
	**Leukemia/Solid tumor** n (%)	6 (8.33)/10 (13.89)	6 (20)/11 (33.3)	4.577 (1.84, 51.383)	< 0.001
	**Trauma** n (%)	3 (4.17)	0 (0)	0.557 (0.547, 0.567)	0.553
	**Sepsis** n (%)	65 (90.27)	25 (83.33)	0.538 (0.156, 1.855)	0.321
	**Peritonitis** n (%)	8 (11.11)	8 (26.67)	2.909 (0.975, 8.68)	0.049
	**Cardiovascular disease** n (%)	8 (11.11)	5 (16.67)	1.35 (0.401, 0.454)	0.627
	**Hepatic disorder** n (%)	5 (6.94)	2 (6.67)	0.814 (0.149, 4.462)	0.813
	**Others** n (%)	10 (13.89)	5 (16.67)	1.24 (0.385, 3.994)	0.718
WBC count(*10^9^/L)	**M(Q1,Q3)**	7.99 (5.81, 13.62)	5.1 (1.64, 14.78)		0.046
Neutropenia(*10^9^/L)	**M(Q1,Q3)**	4.88 (3.51, 9.1)	3.37 (0.61, 11.05)		0.158
CRP (mg/L)	**M(Q1,Q3)**	37 (10, 74)	27 (16, 94.5)		0.406
PCT (ng/L)	**M(Q1,Q3)**	0.48 (0.18, 5.08)	1.56 (0.53, 8.27)		0.066
Lactate (mmol/L)	mean ± SD	3.12 ± 2.9	3.09 ± 2.42		0.955
PRISM III	mean ± SD	6.73 ± 5.19	8.3 ± 4.76		0.183
Shock	n (%)	18, 25%	16, 53.33%	3.429 (1.403, 8.382)	0.006*
PaO_2_/FiO_2_	mean ± SD	269 ± 113.55	220.96 ± 150.16		0.139
Exposure factors					
	**Steroid** n (%)	49 (68.06)	21 (70)	1.619 (0.662, 3.963)	0.289
	**Immune suppressor** n (%)	11 (15.27)	8 (26.67)	2.017 (0.718, 5.665)	0.263
	**Length of hospital day (day,M(Q1,Q3))**	24 (14, 48.75)	31.5 (24.75, 41.5)		0.605
	**Length of PICU day (day,M(Q1,Q3))**	14 (8,23.5)	22.5 (13.25, 41)		0.459
	**Length of broad-spectrum antibiotic therapy (day,M(Q1,Q3))**	22 (12, 43)	31.5 (24.75, 41.5)		0.123
	**Length of mechanical ventilation (day, M(Q1,Q3))**	6 (1, 15)	10 (6, 25)		0.02
	**Length of CRRT/ECMO(h, M(Q1,Q3))**	0 (0, 0)	0 (0, 6)		0.02
	**Central vein catheter** n (%)	69 (95.83)	30 (100)	1.435 (1.26, 1.634)	0.256
	**Arterial catheter** n (%)	58 (80.56)	29 (96.67)	7.0 (0.877, 55.872)	0.036
	**Intra trachea intubation** n (%)	57 (79.17)	29 (96.67)	7.632 (0.96, 60.662)	0.027
	**Operation** n (%)	31 (43.06)	20 (66.67)	2.645 (1.085, 6.448)	0.03

*p < 0.01

### Organ function and immune status of MDR/XDR *Acinetobacter baumannii* infected patients

Table 2 listed the organ function of patients at diagnose of MDR/XDR *Acinetobacter baumannii* infection, as the concern of the importance of immunologic function in the process of anti-inflammatory pathophysiological, the indicators of immune function as well as serum cytokine levels were recorded in Table 3. It seems that no significant differences lies in the organ functions before they developed MDR/XDR *Acinetobacter baumannii* infection, but non-survival patients appeared to have a lower NK cell activity (6.2 ± 3.61% vs. 9.15 ± 6.21%, *P* = 0.029), higher CD4^+^ T cell ratio (39.67 ± 12.18% vs. 32.66 ± 11.44%, *P* = 0.039), at the same time, a higher serum level of interlukin-8 (IL-8, 15.25 (1.62, 47.22)pg/mL vs. 0.1 (0.1, 22.99)pg/mL, *P* = 0.01) was noticed. The results from logistic regression showed that the risk factors for death among MDR/XDR *Acinetobacter baumannii* infection including high level of BUN/ALB and renal dysfunction (Table 4).

**Table 2:** Organ function of patients at diagnose of MDR/XDR Acinetobacter baumannii infection

Organ	Survivors	Nonsurvivors	P value
Cardiovascular			
MAP(mmHg) (mean ± SD)	64.02 ± 12.99	58.13 ± 15.21	0.052
EF(%)(mean ± SD)	65.14 ± 7.06	66.78 ± 3.09	0.251
CI(L/min.m2) ((M(Q1,Q3))	3.8 (3.55, 4.25)	4 (3, 6.1)	0.342
LA(mmol/L) (mean ± SD)	3.12 ± 2.9	3.09 ± 2.42	0.955
Respiratory			
PEEP(cmH_2_O) (mean ± SD)	4.84 ± 1.31	6.24 ± 2.02	< 0.001*
Tidal volume(mL/kg) (mean ± SD)	8.11 ± 0.92	7.98 ± 0.83	0.571
PaO_2_/FiO_2_ (mean ± SD)	269 ± 112.76	269 ± 113.55	0.084
Plateau pressure (cmH_2_O) ((M(Q1,Q3))	10 (9, 12)	11 (10, 13)	0.684
Hepatic			
TBIL(mmol/L) ((M(Q1,Q3))	6.26 (4.14, 9.76)	10.86 (6.64, 18.85)	0.443
ALT(U/L) ((M(Q1,Q3))	25.5 (14, 48)	20 (11.75, 34.75)	0.7
ALB(g/L) ((M(Q1,Q3))	33.68 (30.95, 37.35)	31.88 (27.96, 34.65)	0.027
Renal			
BUN(mmol/L) ((M(Q1,Q3))	3.25 (2.3, 5.53)	5.65 (3.45, 9.43)	0.002*
Cr(umol/L) ((M(Q1,Q3))	22 (17, 36.25)	21 (17.75, 33.25)	0.391
BUN/ALB	0.17 (0.09, 0.27)	0.09 (0.06, 0.16)	0.001*
Nervous			
Glasgow((M(Q1,Q3))	12 (8.25, 13)	10.5 (7,12)	0.152
Gastric-intestine			
Intra-abdominal pressure(cmH_2_O) (mean ± SD)	7.46 ± 4.15	8.95 ± 5.01	0.227

*p < 0.01

**Table 3:** The immunologic function and serum cytokines levels in the patients

Characteristics	Survivors	Nonsurvivors	P value
NK (%) (mean ± SD)	9.15 ± 6.21	6.2 ± 3.61	0.029
IgG(g/L) (mean ± SD)	10.37 ± 4.59	10.56 ± 4.84	0.78
IgA(g/L) (mean ± SD)	1.01 ± 0.92	0.94 ± 0.86	0.77
IgM(g/L) (mean ± SD)	1.03 ± 0.65	0.66 ± 0.45	0.02
CD4^+^(%) (mean ± SD)	32.66 ± 11.44	39.67 ± 12.18	0.039
CD8^+^(%) (mean ± SD)	28.72 ± 13.83	31.38 ± 10.29	0.43
CD19^+^(%) (mean ± SD)	31.38 ± 10.29	23.74 ± 17.18	0.17
IL-6(pg/mL) (M(Q1,Q3))	0.1 (0.1, 22.25)	323.86 (0.1, 733.62)	0.14
IL-8(pg/mL) (M(Q1,Q3))	0.1 (0.1, 22.99)	15.25 (1.62, 47.22)	0.01*
IL-10(pg/mL) (M(Q1,Q3))	6.05 (0.1, 21.81)	16.13 (0.1, 55.07)	0.22

*p < 0.01

**Table 4:** Logistic regression of risk factors

variable	OR (95%CI)	P value
BUN/ALB	107.893 (1.425–870.574)	0.005*
Cr (mmol/L)	0.934 (0.890–0.981)	0.007*

P < 0.05*

### Drug resistance results of MDR/XDR *Acinetobacter baumannii*

**Fig. 2:**
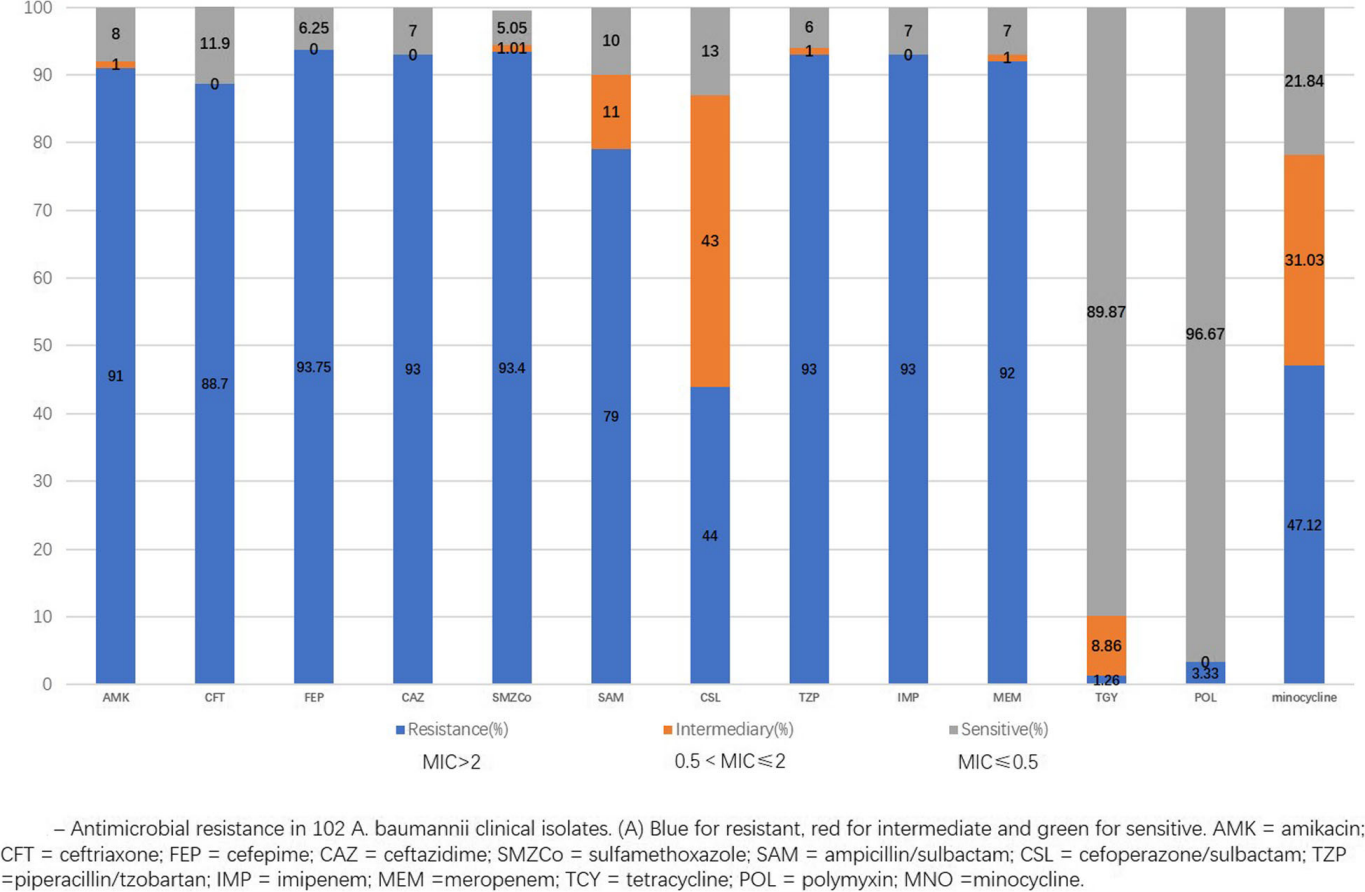
Drug resistance results of MDR/XDR Acinetobacter baumannii in 102 clinical isolates

Bacteriological findings are summarized in Fig. 2. (Antimicrobial resistance in 102 A. baumannii clinical isolates). MDR/XDR *Acinetobacter baumannii* was resistant to carbapenems, aminoglycosides, most cephalosporins and sulfa drugs. Their resistance rates were more than 75%. Only tigecycline, polymyxin had a high sensitivity to MDR/XDR *Acinetobacter baumannii* (tigecycline sensitivity 89.87%, polymyxin sensitivity 96.67%). The resistance rate of cefoperazonesulbactam was 44% and its intermediate rate was 43%.

### Outcomes

A total of 102 patients with *Acinetobacter baumannii* infection were enrolled. All the patients received active antibiotic treatment within 7 days. Regarding treatment given within 7 days, based on the in vitro susceptibility test, cefoperazone/sulbactam, tigecycline, polymyxin were the recommended antimicrobial agents for XDR *Acinetobacter baumannii* eradication. The overall mortality rate was 29.4% (30/102), while the *Acinetobacter baumannii*-associated mortality rate was 16.7% (17/102, 12 bloodstream infections, 4 meningitis and 1 intra-abdominal infection).

## Discussion

*Acinetobacter baumannii*, an aerobic, gram-negative bacillus which is widely distributed in nature, is notorious for its remarkable Ability to acquire antibiotic resistance. As a result, it causes persistent hospital-acquired infections [1]. In our present study, the incidence density of MDR/XDR- *Acinetobacter baumannii* at our facility was 0.48 cases/1000 patient-days, which approximated to the incidence of 0.47 cases/1000 patient-days from reported in ICU patients [19] but lower than the incidence of another PICU that literature reports [2]. Patients in PICU always characterized by more severe underlying diseases, immune dysfunction and more complex medical history, all of these conditions will cause severe infection of *Acinetobacter baumannii* while MDR/XDR *Acinetobacter baumannii* strains appeared.

From our single-center data, ventilator-associated pneumonia (VAP) occupied the major complication in pediatric critically ill patients who MDR/XDR *Acinetobacter baumannii* infection with an incidence rate of 54.9% (56/102). Both blood stream and CNS infection caused relative high mortality, in our research, 12 in 28 bloodstream infection patients and all 4 meningitis patients died. Due to increased invasive operations as well as the severity of primary conditions of critically ill patients, the overall incidence of MDR/XDR *Acinetobacter baumannii* infection increased.

MDR/XDR *Acinetobacter baumannii* is resistant to most pediatric antimicrobial agents, including penicillins, most cephalosporins, carbapenems, aminoglycosides, and sulfa drugs. XDR *Acinetobacter baumannii* tends to develop resistance to multiple antimicrobial agents through degrading enzymes targeting β- lactams, increasing the expression of Ampc enzyme, modifying enzymes targeting aminoglycosides and alteration to the binding sites for quinolones, producing OXA-23 carbapenem enzyme, decreasing the expression of outer membrane pore channel protein, efflux pump system hyperactivity, and loss of PBPs. Additionally, MDR/XDR *Acinetobacter baumannii* could induce drug resistance through plasmid integration, while causing multiple drug resistance plasmids [20, 21]. In our study, the susceptibility results showed that the drug resistance rates of MDR/XDR *Acinetobacter baumannii* to beta-lactam antibiotics were more than 75% except for cefoperazone/sulbactam (42%). When it came to the in vitro activities of beta-lactamase inhibitors, sulbactam was superior to clavulanic acid and tazobactam. Sulbactam has good intrinsic antimicrobial activity against multidrug-resistant acinetobacter strains at concentrations readily achievable in human serum [22]. As the contribution of sulbactam, another antibiotic contains sulbactam-ampicillin/sulbatan, showed lower drug resistance rate (79%) than other beta-lactam antibiotics. During the period of neither tigecycline nor polymyxin was available, a combination therapy of cefoperazone/sulbactam or fosfomycin and carbapenems was applied.

Since the safety of children’s medication has been put in an important place, our choice of antibiotics faced a lot of limitations. Although no evidence in the literature that combination therapy is prior to single drug for infection with MDR/XDR *Acinetobacter baumannii*, some in vitro studies have shown that certain drug combinations are synergistic [23, 24]. In the study by Singkham-In U et al [25], the combination of 1× MIC of imipenem (16–64 mg/L, and 128 mg/L of isolate A10) and 1× MIC of fosfomycin (128–256 mg/L) showed synergism and bactericidal effect against most *Acinetobacter baumannii* isolates. Fosfomycin, when combined with imipenem, may enhance the inhibition of bacterial cell wall synthesis. So, from this point of view, the combination of fosfomycin and carbapenems in the past few years benefited a number of our patients.

*Acinetobacter baumannii* was transported together by infiltrating neutrophils. Go Kamoshida et al. [26] found that *Acinetobacter baumannii* exploits human neutrophils by adhering to and inducing IL-8 release for bacterial portage. *Acinetobacter baumannii* stimulation IL-8 plays a critical role in enhancing the migration of *Acinetobacter baumannii* -adhering neutrophils, the migration of *Acinetobacter baumannii* was suppressed when the infiltration of neutrophils was suppressed by inhibiting IL-8 [27]. Through Toll-like receptor 4 (TLR4) and CD14, *Acinetobacter baumannii* lipopolysaccharide leads the production of the neutrophil chemotactic factor IL-8 and the proinflammatory cytokine TNF-α [28]. In our MDR/XDR *Acinetobacter baumannii* infected patients, a higher serum level of IL-8 (15.25 (1.62, 47.215)pg/mL vs. 0.1(0.1, 22.99)pg/mL) was detected in the non-survival group, this phenomenon might explain that *Acinetobacter baumannii* in this group seems to spread throughout the body more easily.

Studies have shown that purified TLR2 ligands from *Acinetobacter baumannii* are immunostimulatory [29]. CD4^+^ T cells play important role in the Th1/Th2 paradigm, participate in inflammation [30], AbOmpA of *Acinetobacter baumannii* (AbOmpA) is a major porin protein in the outer membrane and is partly responsible for apoptosis of eukaryotic cells. Jun SikLee et al [31] co-cultured CD4+ splenic T cells with AbOmpA-treated DCs, and found AbOmpA directs CD4^+^ T cell differentiation towards a Th1 response. Most biofilms provide a mechanical barrier to phagocytosis much like a capsule. A small number of bacteria become biologically active by migration from the biofilm or by shearing forces that remove small clumps of the biofilm. These bacteria released from the biofilm can induce not only host responses but also act as the seeding colony for the establishment of another infectious focus [32]. In our research, the patients in non-survival group appeared a higher CD4^+^ T cell ratio than survival patients, which revealed that a persistent neutrophil activation and accumulation in tissue that caused inflammation spread.

Critically ill patients had increased oxygen consumption, when these patients accompanied with capillary leak syndrome, they often had hydration status resulting in increasing reabsorption of urea by the kidneys, and reduction of ALB, elevation of BUN level can be frequently observed. Current studies highlighted BUN/ALB level as a predictor of 30-day mortality in a large sample of adult patients with hospital acquired pneumonia [33], and in our research, we also find that high BUN/ALB level associate with worse clinical outcome (0.09(0.06, 0.16)vs. 0.17(0.09, 0.27), *p* = 0.001), which coincide with the results in adult patients.

Our study had several limitations. First, because data were collected retrospectively from medical records, some parameters had to be inferred from the charts. Susceptibility testing had limitations, fosfomycin was not included, which resulted in a lack of antibiotic susceptibility evidence when we made treatment decisions.

In conclusion, our study suggests that MDR/XDR- *Acinetobacter baumannii* is an important opportunistic pathogen that causes nosocomial infection in PICU with a rather high mortality. The incidence increased these years. Bloodstream and central nervous infection accounted for high risk of death. Increased invasive operations, IL-8 releasing, and persistent neutrophil activation contributed to the risk of death.

## Conclusion

MDR/XDR *Acinetobacter baumannii* infection is a serious concern in pediatric patients with high mortality. Bloodstream and central nervous system infection accounted for high risk of death. Acute kidney injury is associated with high risk of mortality.

## Data Availability

Our present study was a retrospective observational study. All the data were obtained from medical records of patients. The datasets used and/or analysed during the current study are available from the corresponding author on reasonable request, but the identifying/confidential patient data would not be shared.

## Abbreviations

MDR: Multidrug resistant
XDR: Extensively drug resistant
PDR: Pandrug resistant
PICU: Pediatric intensive care unit
CSF: Cerebrospinal fluid
CLSI: Clinical and Laboratory Standards Institute
PRISM III: Pediatric risk of mortality III
AKI: Acute kidney injury
ALT: Alanine transaminase
CI: Cardiac index
BUN: Blood urea nitrogen
Cr: Creatinine
VAP: Ventilator-associated pneumonia
CRRT: Continuous renal replacement therapy
ECMO: Extracorporeal membrane
Lac: Lactate
EF: Left ventricular ejection fraction
PaO_2_/FiO_2_: The ratio of the partial pressure of oxygen in arterial blood (PaO2) to the inspired oxygen fraction (FiO2)
